# Prevalence and determinants of obesity - a cross-sectional study of an adult Northern Nigerian population

**DOI:** 10.1186/1755-7682-4-10

**Published:** 2011-03-01

**Authors:** Kolawole W Wahab, Mahmoud U Sani, Bashir O Yusuf, Maruf Gbadamosi, Akeem Gbadamosi, Mahmoud I Yandutse

**Affiliations:** 1 Department of Medicine, University of Ilorin Teaching Hospital, Ilorin, Nigeria; 2 Department of Medicine, Aminu Kano Teaching Hospital, Kano, Kano state, Nigeria; 3 Department of Medicine, Federal Medical Centre, Katsina, Katsina state, Nigeria; 4 Department of Chemical Pathology, Federal Medical Centre, Katsina, Katsina state, Nigeria

## Abstract

**Background:**

Obesity is assuming an epidemic dimension globally. It is important to appreciate factors associated with the disease so that a holistic approach can be taken in tackling the rising burden. The objective of this study was to determine the prevalence of overweight and obesity and the factors independently associated with obesity in an urban Nigerian population.

**Methods:**

A cross-sectional study of 300 healthy adult subjects was conducted in the urban city of Katsina, northern Nigeria. Relevant sociodemographic and clinical information were obtained. Screening for obesity was done using the Body Mass Index while relevant laboratory investigations were conducted. Univariate and multivariate logistic regression analyses were performed to determine the predictors of obesity.

**Results:**

Overweight and obesity was found in 53.3% and 21.0% respectively with a significantly higher prevalence in females compared to males (overweight: 62.0% vs 41.9%, p < 0.001; obesity: 29.8% vs 9.3%, p < 0.001). In univariate analysis, the odds of obesity were higher in women and in the presence of hypertension, hypercholesterolaemia and hyperuricaemia. However, in multivariate analysis, factors independently associated with obesity were female sex (OR 6.119, 95% CI 2.705-13.842, p < 0.001), hypercholesterolaemia (OR 2.138, 95% CI 1.109-4.119, p = 0.023) and hyperuricaemia (OR 2.906, 95% CI 1.444-5.847, p = 0.003).

**Conclusion:**

There is a high prevalence of obesity in northern Nigeria and women are significantly more affected. The high prevalence is independently associated with female sex, hypercholesterolaemia and hyperuricaemia. Public health education is urgently needed in order to reduce this burden and prevent other non-communicable cardiovascular disorders.

## Background

Although it used to be regarded as a disease of industrialized countries, obesity, commonly defined as a body mass index (BMI) ≥ 30 kg/m^2 ^is assuming an epidemic dimension globally [1]. Aside from being a potentially modifiable cardiovascular disease (CVD) risk factor on its own, this non-communicable disease predisposes to other CVD risk factors such as diabetes mellitus, hypertension, dyslipidaemia and metabolic syndrome among others [2]. Based on measured weight and height, the prevalence of obesity in the United States was found to be 30.5% in a survey conducted from 1999 to 2000 while in the United Kingdom, the prevalence is 23% among men and 24% among women [3,4]. In the West African countries of Ghana and Republic of Benin, obesity is found in 13.6% and 18% respectively among adults [5,6] while Abubakari et al reported a prevalence of 10% in the West African sub-region with the odd of being obese being 3.2 among urban women compared to men [7]. Obesity is becoming increasingly more prevalent in many African and other developing countries with nutritional transition as a result of urbanization, adoption of western lifestyles and demographic transition being implicated for the upsurge [8,9].

Nigeria is witnessing both demographic and epidemiologic transitions and these could be some of the possible reasons why the prevalence of non-communicable diseases is increasing. There is also a general misconception in Nigeria that obesity is a sign of affluence [8]. In a cross-sectional study in southwestern Nigeria, Ojofeitimi et al [8] found that 21.2% of their respondents were obese while Kadiri & Salako found obesity in 21% and 28% of males and females respectively in a study of 146 middle-aged Nigerians [10].

Although there have been studies on prevalence of obesity in Nigeria, there is a paucity of information on factors associated with it. Establishment of associated factors will be potentially useful in the holistic approach to the prevention of the rising prevalence of obesity and other non-communicable cardiovascular diseases. The aims of this study were therefore to determine the prevalence of overweight and obesity and also determine the factors that would independently predict obesity among apparently healthy adult Nigerians in the north western city of Katsina.

## Methods

This was a descriptive cross-sectional study carried out in Katsina city, in the northwestern region of Nigeria between March and May, 2006. This urban city, which has a total population of 459,022 based on 2006 census figures, is composed majorly of Hausa and Fulani tribes while other Nigerian tribes are found in minority. The predominant occupation of the people is farming while many educated elite work in government or private establishments.

Based on a prevalence of 21.2% from a previous study in the southwestern part of the country [8] and a level of precision of 5%, the desired sample size was calculated to be 257. Subjects were consecutively recruited using convenience sampling technique till the desired sample size was achieved. The recruited respondents were members of the public, hospital staff and relations of inpatients who were requested to come for a free screening after a health education on the need for prevention of CVD risk factors was given to them by the investigators. Screening was conducted at the Federal Medical Centre, Katsina which is a tertiary hospital that serves as a referral centre for all primary and secondary healthcare facilities in Katsina state. Subjects gave their voluntary informed consent to participate in the study while the study protocol was approved by the research and ethics committee of the hospital. In a standardized manner, information was obtained on relevant sociodemographic characteristics like age, sex, current history of alcohol or tobacco use, hypertension and diabetes mellitus with the aid of an interviewer-administered semi-structured questionnaire.

### Anthropometric and blood pressure measurements

All anthropometric measurements were made by two trained research assistants. Weight was taken to the nearest 0.1 kilogramme using a weighing scale while for measurement of height, a stadiometer was used. Body mass index (BMI) was then calculated from weight (in kilogrammes) divided by a square of the height (in metres). Blood pressure was measured in the left arm in the sitting position with the aid of a mercury sphygmomanometer using the auscultation method. The systolic blood pressure was recorded at phase I Korotkoff sounds while the diastolic blood pressure was recorded at phase V Korotkoff sounds. All measurements were taken twice by the same person and the average of the two readings was taken as final.

### Biochemical analysis

After 10-12 hours of overnight fast, venous samples were obtained from the subjects. The blood was immediately centrifuged while the serum obtained was analyzed for glucose, total cholesterol and uric acid concentrations. Serum glucose, total cholesterol and uric acid estimations were done at the hospital's central laboratory by the same laboratory technologist.

### Definition of terms

BMI (kg/m^2^) was categorized using the World Health Organization (WHO) definitions: BMI of 18.5-24.9 kg/m^2 ^was used as the reference (normal BMI), 25-29.9 kg/m^2 ^was used to define overweight while ≥30 kg/m^2 ^was used for definition of obesity. Obesity was further sub classified into class 1 (30-34.9 kg/m^2^), class 2 (35-39.9 kg/m^2^) and class 3 (≥40 kg/m^2^) [11]. Hypertension was defined as systolic blood pressure ≥140 mmHg and/or diastolic blood pressure ≥90 mmHg [12]. Diabetes mellitus was defined as a fasting plasma glucose ≥7 mmol/L while hypercholesterolaemia and hyperuricaemia were defined as ≥200 mg/dl and ≥420 μmol/L respectively [13].

### Statistical analysis

Data was analyzed using the Statistical Package for the Social Sciences version 17.0 (SPSS Inc.). Means and standard deviations were obtained for relevant variables while frequency tables were also generated. For comparison of categorical variables, the Chi-square test was used while for continuous variables, the student's t-test was used. Overall and gender prevalence of overweight and obesity was determined by generation of frequency tables. Predictors of obesity were determined using a univariate binary logistic regression model. The predictors assessed were gender, level of education (dichotomized into <12 years or ≥12 years), monthly income (<N50,000.00 (~$340.00) and ≥N50,000.00), history of smoking or alcohol use, and presence or absence of systolic and diastolic hypertension, diabetes mellitus, hypercholesterolaemia and hyperuricaemia. To determine the independent predictors of obesity and eliminate potential confounders, a multivariate logistic regression analysis was done using backward elimination technique. The results of regression analyses were reported as odds ratios with their respective 95% confidence intervals. Using a 2-tailed test, statistical significance was set at *p *< 0.05.

## Results

### Sociodemographic and clinical characteristics of the respondents

Out of the 321 eligible respondents screened, 300 (93.5%) with a mean age of 37.6 ± 10.6 years had complete information. Overall, 60.3% of the respondents had at least 12 years of formal education with males being better educated compared to females (75.1% vs 49.1%, p < 0.001). Males also tended to have statistically higher monthly income, history of cigarette smoking and alcohol consumption while history of hypertension was more common in females (20.5% vs 10.1%, p = 0.02). All these are presented in table 1.

**Table 1:** Sociodemographic and clinical characteristics of the respondents

Variable	Overall (n = 300)	Males (n = 129)	Females (n = 171)	*p *value
Mean age ± SD, yrs	37.6 ± 10.6	38.0 ± 9.7	37.2 ± 11.2	0.54
Years of education				
0-12	119 (39.7)	32 (24.9)	87 (50.9)	<0.001*
≥12	181 (60.3)	97 (75.1)	84 (49.1)	
Monthly income, N				
<50,000	228 (76.0)	81 (62.8)	147 (86.0)	<0.001*
≥50,000	72 (24.0)	48 (37.2)	24 (14.0)	
Cigarette Smoking	14 (4.7)	11 (8.5)	3 (1.8)	0.006*
Alcohol intake	17 (5.7)	14 (10.9)	3 (1.8)	<0.001*
History of HTN	48 (16.0)	13 (10.1))	35 (20.5)	0.02*
Mean SBP ± SD, mmHg	120.4 ± 21.1	117.9 ± 18.9	122.3 ± 22.4	0.07
Mean ± SD DBP, mmHg	78.9 ± 13.5	77.4 ± 11.8	80.1 ± 14.6	0.10
History of DM	10 (3.3)	4 (3.1)	6 (3.5)	0.90
Mean ± SD FPG, mg/dl	82.9 ± 20.9	82.9 ± 15.4	82.9 ± 24.2	0.99

Except otherwise stated, values are n (%). N = Naira; HTN = hypertension; SBP = systolic blood pressure; DBP = diastolic blood pressure; DM = diabetes mellitus; FPG = fasting plasma glucose.

*p < 0.05

### Prevalence of overweight and obesity

As shown in table 2, 160 (53.3%) of the respondents were overweight (BMI ≥25 kg/m^2^) while 63 (21.0%) were obese (BMI ≥30 kg/m^2^). The prevalence of overweight was significantly higher among females compared to male respondents (62.0% vs 41.9%, p < 0.001); likewise, obesity was more common in females (29.8% vs 9.3%, p < 0.001). All classes of obesity were also more common in female respondents.

**Table 2:** Overall and gender prevalence of overweight and obesity

BMI†	Male (n = 129); n (%)	Female (n = 171); n (%)	Overall (n = 300); n (%)	*p *value
Overweight	54 (41.9)	106 (62.0)	160 (53.3)	0.0005*
Obesity	12 (9.3)	51 (29.8)	63 (21.0)	0.00002*
*Class 1*	10 (7.8)	32 (18.7)	42 (14.0)	0.007*
*Class 2*	2 (1.6)	12 (7.0)	14 (4.7)	0.026*
*Class 3*	0 (0.0)	7 (4.1)	7 (2.3)	0.05

†Body mass index; *p < 0.05

### Predictors of obesity (tables 3 and 4)

**Table 3:** Univariate binary logistic regression analysis to determine predictors of obesity

Predictor	Odds ratio	95% CI	p value
Female sex	4.972	2.410-10.258	<0.001*
<12 years of formal education	0.845	0.477-1.496	0.563
Income > N50, 000	0.919	0.472-1.790	0.804
Smoking	0.287	0.037-2.239	0.234
Alcohol consumption	1.214	0.337-4.365	0.767
Systolic BP ≥140 mmHg	2.194	1.143-4.211	0.018*
Diastolic BP ≥90 mmHg	2.175	1.147-4.127	0.017*
Hypercholesterolaemia	2.590	1.432-4.687	0.002*
Hyperuricaemia	1.944	1.072-3.524	0.029*

*p < 0.05

**Table 4:** Multivariate logistic regression analysis to determine independent predictors of obesity

Predictor	Odds ratio	95% CI	p value
Female sex	6.119	2.705-13.842	<0.001*
Systolic BP ≥140 mmHg	1.452	0.543-3.881	0.457
Diastolic BP ≥90 mmHg	1.414	0.542-3.693	0.479
Hypercholesterolaemia	2.138	1.109-4.119	0.023*
Hyperuricaemia	2.906	1.444-5.847	0.003*

*p < 0.05

In univariate analysis, the identified predictors of obesity were female gender (OR 4.972, 95% CI 2.410-10.258, p <0.001), systolic hypertension (OR 2.194, 95% CI 1.143-4.211, p = 0.018), diastolic hypertension (OR 2.175, 95% CI 1.147-4.127, p = 0.017), hypercholesterolaemia (OR 2.590, 95% CI 1.432-4.687, p = 0.002) and hyperuricaemia (OR 1.944, 95% CI 1.072-3.524, p = 0.029). However, when all these significant variables from univariate analysis were entered into a multivariate logistic regression model, the independent predictors of obesity were female sex (OR 6.119, 95% CI 2.705-13.842, p < 0.001), hypercholesterolaemia (OR 2.138, 95% CI 1.109-4.119, p = 0.023) and hyperuricaemia (OR 2.906, 95% CI 1.444-5.847, p = 0.003).

## Discussion

This cross-sectional study among apparently healthy adult Nigerians shows the prevalence of overweight (BMI ≥25 kg/m^2^) to be 53.3% with a statistically higher prevalence among females compared to males (62.0% vs 41.9%). For obesity, the overall prevalence was 21% with a male to female ratio of approximately1:3. Also, grades 1 and 2 obesity were significantly higher among females compared to males while the only seven subjects with grade 3 obesity were females. After controlling for other confounding variables, factors independently associated with obesity were female gender, hypercholesterolaemia and hyperuricaemia.

When compared to the findings from other African countries, the prevalence of obesity obtained in this study is similar to the 18% that was reported in a study among urban dwellers in the Republic of Benin but lower than the 13.6% reported in Ghana [5,6]. The latter study was however conducted among two urban and one rural communities and this selection method could have accounted for the difference in the prevalence. An earlier cross-sectional study in the southwestern part of Nigeria also found obesity to be present in 21.2% of the subjects [8]. The overall prevalence in our study is however lower than that reported in the US where the prevalence of obesity increased from 22.9% in the late 1980 s to early 1990 s to 30.5% between 1999 and 2000 [3]. The possible explanation for the difference between the US study and ours could be because Nigeria is just undergoing both nutritional and epidemiologic transitions. In many cities, fast food outlets are rapidly springing up with high patronage, leading to the consumption of energy-dense foods which is probably fuelling the high prevalence. Except a pro-active approach is taken, the situation may even get worse because there are presently no measures in place to stem the tide [14].

Results of multivariate analysis show that women are at about 6.1 folds higher risk of being obese compared to men and even after adjusting for gender and other confounding variables, the odds of obesity are still significantly associated with hypercholesterolaemia and hyperuricaemia. The association with female gender is not surprising given their almost 3 times higher prevalence of obesity when compared to men. The odds ratio obtained in this study is higher than the 3.16 (95% CI 2.51-3.98) reported for West African urban women compared to men in a metaanalysis by Abubakari et al [7] possibly because the latter was a pooled analysis of studies in the sub-region. A higher obesity prevalence of 28% in women compared to 21% in men has also been reported from a study of respondents in their middle ages in the southwestern part of Nigeria [10]. Our finding is also quite similar to what has been documented in other parts of Africa [5,6,15,16]. The plausible explanation for the higher odds of obesity among our female respondents could be a superimposition of sedentary lifestyle on the nutritional transition being witnessed in the country although we did not obtain information on physical activity in our study. However, in the northern part of the country where this study was conducted, a lot of women are engaged in sedentary jobs if they work at all. Engagement in regular physical exercise is also not a common thing in the general population and this is even worse in women. Indeed, in a study of risk factors for diabetes mellitus in a non-diabetic population in northern Nigeria, Puepet et al reported that 50% of their respondents were physically inactive and the prevalence of obesity using waist: hip ratio was also approximately 3 times higher in females [17]. The perception of obesity as a sign of affluence by many people (especially women) in this part of the world could have also contributed. Ojofeitimi et al found that in spite of the higher education of their subjects in a university community in southwestern Nigeria, many of the respondents believed that being obese gives respect and that it is a sign of good living [8].

That hypercholesterolaemia and hyperuricaemia independently increase the odds of obesity 2.1 and 2.9 folds respectively further buttress the fact that cardiovascular disease risk factors tend to cluster. Obesity is known to increase the risk of dyslipidaemia and hypertension [18], and in univariate analysis, we found that both systolic and diastolic hypertension were significantly associated with obesity although this effect was lost in multivariate analysis. Thomas et al showed that the prevalence of hypercholesterolaemia, hypertension and diabetes progressively increased with BMI irrespective of the gender of their subjects [19]. A recent study in Tunisia, North Africa also showed a clustering of cardiovascular disease risk factors like impaired fasting glucose, dyslipidaemia and hyperuricaemia among obese subjects [20].

The main strength of our study is the fact that apart from studying the prevalence of overweight and obesity, we were also able to assess the independent predictors of obesity in a multivariate analysis so that a holistic approach can be taken in tackling the rising burden of CVD in the country. We however appreciate that the study had some limitations which may not make the results generalizable to the entire population. The cross-sectional nature of the study and the non-probability sampling technique used could have possibly excluded some respondents. However, as regards the latter situation, we tried to reduce selection bias by including all consecutively consenting respondents. It is also possible that ethnicity, level of physical activity and eating pattern could have influenced the results observed; however since we did not obtain data on these variables, our results should be interpreted with caution because these are some of the factors that could affect body weight. In spite of these limitations, we believe that our findings can serve as an eye opener to the brewing burden of obesity and can also serve as a template for a proper community-based study.

## Conclusions

From the results of this study, it could be postulated that there is an urgent need to mount an intensive public health education with the aim of reducing the present unacceptably high prevalence of overweight and obesity in Nigeria, especially in females. Healthy living in terms of consumption of fruits and vegetables, regular aerobic exercises and discouragement of consumption of calorie-dense diets are some of the issues that should be addressed in educating the populace on this avoidable epidemic. If we consider the fact that the battle against communicable diseases like malaria, multi-drug resistant tuberculosis and HIV/AIDS is still far from being won, all efforts should be made to stem the tide of the rising prevalence of overweight and obesity so that the burden of other related non-communicable cardiovascular disorders can be reduced and the available meager resources can be properly channeled.

## Competing interests

The authors declare that they have no competing interests.

## Authors' contributions

KWW and MUS conceived and designed the study. BOY, MG and AG were involved in initial literature search. All authors were involved in data collection while MIY did all laboratory analyses. KWW drafted the initial manuscript while MUS read the initial manuscript for major intellectual input. All authors read and approved the final draft.
